# Dapagliflozin: A Promising Strategy to Combat Cisplatin-Induced Hepatotoxicity in Wistar Rats

**DOI:** 10.3390/biology13090672

**Published:** 2024-08-29

**Authors:** Shakta Mani Satyam, Laxminarayana Kurady Bairy, Abdul Rehman, Mohamed Farook, Sofiya Khan, Anuradha Asokan Nair, Nirmal Nachiketh Binu, Mohamed Yehya, Mohammed Moin Khan

**Affiliations:** 1 Faculty of Pharmacology, RAK College of Medical Sciences, RAK Medical and Health Sciences University, Ras Al Khaimah 11172, United Arab Emirates; kurady@rakmhsu.ac.ae; 2 Faculty of Pathology, RAK College of Medical Sciences, RAK Medical and Health Sciences University, Ras Al Khaimah 11172, United Arab Emirates; rehman@rakmhsu.ac.ae; 3 RAK College of Medical Sciences, RAK Medical and Health Sciences University, Ras Al Khaimah 11172, United Arab Emirates; mohamed.21901055@rakmhsu.ac.ae (M.F.); sofiya.21901051@rakmhsu.ac.ae (S.K.); anuradha.21901047@rakmhsu.ac.ae (A.A.N.); nirmal.21901034@rakmhsu.ac.ae (N.N.B.); mohamed.21901028@rakmhsu.ac.ae (M.Y.); mohammed.21901064@rakmhsu.ac.ae (M.M.K.)

**Keywords:** sodium-glucose co-transporter-2 inhibitors, hepatotoxicity, cisplatin, chemotherapy, chemoprotectant, repurposing, antidiabetics

## Abstract

**Simple Summary:**

Repurposing the diabetes drug dapagliflozin and the natural agent silymarin, this study tackled the liver damage caused by the chemotherapy drug cisplatin. Thirty female Wistar rats were treated with cisplatin alone, silymarin, dapagliflozin, or their combination for 45 days. The results were promising; cisplatin-induced liver damage, marked by elevated ALT, AST, and TB levels, and decreased TP and albumin levels, was significantly mitigated by dapagliflozin and silymarin. The combination of both showed promising results as well, restoring the liver enzyme levels and tissue structure. This novel approach not only highlights the powerful hepatoprotective effects of dapagliflozin and silymarin but also opens new avenues for safer chemotherapy treatments. Further mechanistic research could turn these findings into clinical practice, offering new hope for cancer patients undergoing cisplatin-based therapy.

**Abstract:**

Recognizing the challenges posed by chemotherapy, specifically the hepatotoxic effects of drugs like cisplatin, this study aimed to examine the hepatoprotective potential of dapagliflozin to mitigate cisplatin-induced hepatotoxicity in a rat model. This study focused on repurposing drugs such as dapagliflozin and natural agents like silymarin as potential interventions to address cisplatin-induced hepatotoxicity. Thirty adult female Wistar rats were distributed into five groups and treated with cisplatin alone, silymarin, dapagliflozin, or a combination of dapagliflozin and silymarin accordingly for 45 days. Body weight, fasting blood glucose levels, liver function tests, and histopathological analysis were conducted to evaluate the hepatoprotective effects. Cisplatin-induced hepatotoxicity significantly (*p* < 0.05) increased the serum levels of ALT, AST, TB, and reduced the TP and albumin levels. Dapagliflozin administration led to significant reductions in ALT, AST, TB, and increased albumin levels. Silymarin demonstrated comparable effects. Combining dapagliflozin and silymarin showed synergistic effects, further reducing the liver enzymes and improving albumin levels. Histopathological examination supported these findings, revealing the restoration of liver structure with dapagliflozin and silymarin treatment. Dapagliflozin and silymarin exhibited substantial hepatoprotective benefits against cisplatin-induced hepatotoxicity in rats. The combination therapy demonstrated synergistic effects, highlighting a potential therapeutic approach for mitigating chemotherapy-induced liver damage. Further research into molecular mechanisms and clinical translation is warranted, offering hope for improved clinical outcomes in cancer patients undergoing cisplatin-based chemotherapy.

## 1. Introduction

Cancer represents a substantial portion of the global disease burden, and predictions suggest a sustained rise in worldwide cancer prevalence for at least the next two decades [1]. Despite progress in the medical understanding and technology that has resulted in better cancer detection and treatment methods, increased survival rates frequently bring about the difficulty of treatment-related toxicities, negatively impacting the health and quality of life of patients [2]. Although the improvement in cancer survival rates is noteworthy, the growing occurrence of treatment-related side effects and associated health issues continues to be a matter of concern.

Cisplatin, a potent chemotherapy drug, is extensively utilized in treating various cancers such as ovarian, testicular, and lung malignancies. Despite its effectiveness, the clinical application of cisplatin is limited due to its adverse effects, particularly hepatotoxicity, which often necessitates dose adjustments or the discontinuation of treatment thus compromising therapeutic outcomes [3]. The liver plays a crucial role in metabolizing and detoxifying substances. The well-known toxic effect of cisplatin is nephrotoxicity [4]. Furthermore, research has indicated that hepatotoxicity is another dose-limiting side effect of cisplatin-based chemotherapy [5]. Additionally, cisplatin can induce DNA damage in the bone marrow and lymphocytes [6]. The generation of ROS causing increased lipid peroxidation and cellular destruction is the mechanism underlying cisplatin-induced toxicity. Furthermore, apoptosis occurs in both cancerous and normal cells [7]. Cisplatin-induced hepatotoxicity is characterized by oxidative stress, inflammation, and apoptosis, mediated by ROS and inflammatory cytokines in hepatocytes [8]. Cisplatin primarily exerts its cytotoxic effects by building covalent DNA adducts and resulting in cell death. Unfortunately, its indiscriminate cytotoxicity can affect normal tissues, including the liver, which is particularly vulnerable due to its role in drug metabolism and detoxification. Cisplatin rapidly penetrates multiple tissues after administration, with increased concentrations in the liver [9]. The cellular uptake of cisplatin occurs through passive transport, a mechanism believed to be its primary mode of entry [9]. Cytochrome P450 2E1 (CYP2E1) in the microsomal ethanol oxidizing system is a major generator of reactive oxygen species (ROS) in the liver and is considered a significant contributor to alcoholic liver disease [10]. Once inside the hepatocyte, cisplatin undergoes metabolism by the cytochrome P450 (CYP450) enzyme complex, particularly CYP2E1, leading to hepatotoxicity [10]. CYP2E1 is also present in trace amounts in the brain, lungs, gastrointestinal tract, kidneys, and lymphocytes [11]. Both in vitro and in vivo studies indicate that elevated levels of CYP2E1 exacerbate cisplatin-induced hepatotoxicity, potentially through increased ROS production and oxidative stress [11]. Cisplatin enters cells primarily through passive transport, which has long been considered the main mechanism for its cellular uptake [9]. Once inside the cells, cisplatin undergoes hepatic metabolism and biotransformation by the cytochrome P450 enzyme complex. Among these, cytochrome P450 2E1 is the most prominent enzyme reported in the literature for its involvement in hepatotoxicity, especially in drugs metabolized by CYP2E1 [10,12]. Cisplatin-induced liver damage is also associated with ROS generation by mitochondria [9]. The mechanisms of cisplatin-induced hepatotoxicity include membrane rigidity; decreased glutathione reduced/oxidized ratio (GSH/GSSG), adenosine triphosphate (ATP), GSH, and nicotinamide adenine dinucleotide phosphate (NADPH) levels; lipid peroxidation; and oxidative damage to cardiolipin and protein sulfhydryl groups [13].

Hepatotoxicity has been observed in patients receiving low doses of cisplatin, likely due to its accumulation in the hepatocytes, resulting in significant toxicity characterized by effacement of the hepatic cords, inflammatory infiltrates, and necrosis [14]. Despite extensive research, strategies to mitigate cisplatin-induced hepatotoxicity remain limited, hampering the therapeutic potential of this widely used chemotherapy drug.

Recent investigations suggest that plant extracts, such as silymarin, obtained from the seeds of *Silybum marianum*, frequently called milk thistle, are utilized in the treatment of liver diseases [15,16]. Research has demonstrated its strong hepatoprotective antioxidant effects, which are achieved by preventing lipid peroxidation [17,18].

The strategy of repurposing existing medications for novel therapeutic applications has become increasingly popular in drug discovery and development. This approach can accelerate the availability of treatments for a range of medical conditions and offers several significant advantages, such as cost savings, shorter development timelines, and enhanced patient outcomes. Developing a new drug from scratch is time-consuming and costly, whereas repurposing existing drugs significantly cuts down on costs and time by leveraging prior safety testing. Since repurposed drugs have already undergone safety testing, they can potentially skip certain preclinical and early clinical development phases, accelerating the availability of treatments, especially in urgent situations. Repurposing drugs also provides comprehensive safety data, streamlining regulatory approval and instilling confidence in their safety profiles. Furthermore, repurposing allows the exploration of alternative uses for medications, potentially uncovering novel therapeutic effects and addressing unmet medical needs, thereby advancing medical science and patient care.

Sodium–Glucose Cotransporter 2 inhibitors (SGLT2i), originally developed to manage type 2 diabetes mellitus (T2DM), have garnered interest for their diverse effects beyond glycemic regulation. Dapagliflozin, an inhibitor of SGLT2, has demonstrated pleiotropic effects in terms of mitigating oxidative stress, apoptosis, and inflammation in various disease models [19]. Although it is best known for its antihyperglycemic benefits, emerging research has revealed its additional advantages, such as weight loss, cardiovascular improvements, and enhanced metabolic parameters [20,21]. The antioxidant capabilities of dapagliflozin, which involve reduced reactive oxygen species (ROS) production and the modulation of calcium (Ca^2+^) influx, in addition to ameliorating inflammation, might be the prime reason for its therapeutic role in mitigating hepatotoxicity caused by cisplatin [22]. Human hepatocellular carcinoma cells (HepG2) are known to have both SGLT-1 and SGLT-2 transporters [23,24]. Evidence suggests that SGLT2i may reduce cell proliferation in various hepatocellular cell lines, partially due to their ability to lower glucose uptake [25,26]. This preclinical investigation aimed to assess the hepatoprotective potential of dapagliflozin against cisplatin-induced hepatotoxicity using a rat model.

By harnessing its antioxidative, anti-inflammatory, and antiapoptotic properties [27], repurposing dapagliflozin could offer a novel approach to mitigate cisplatin-induced liver damage, enhancing the safety and efficacy of cisplatin-based chemotherapy regimens.

## 2. Materials and Methods

### 2.1. Drugs and Reagents

The active pharmaceutical ingredient of silymarin was sourced from Sigma-Aldrich-Merck Limited, Bangalore, India. Dapagliflozin was acquired from AstraZeneca, Mount Vernon, IN, USA. Colorimetric assay kits for ALT (catalogue no. AV8920), AST (catalogue no. AV8910), Total Bilirubin (catalogue no. AV8231), Total Protein (catalogue no. AV8710) and Albumin (catalogue no. AV8201) were obtained from Avicenna Alliance Global, Dubai, UAE. The glucometer and glucose test strips (Manufacturer—Trister, Lewes, DE, USA) were purchased from Life Pharmacy, Ras Al Khaimah, UAE. All laboratory-grade chemicals were obtained through local distributors in the UAE.

### 2.2. Animals

A total of 30 inbred adult female Wistar rats, aged 8 to 10 weeks and weighing between 150 and 200 g, were maintained under standardized conditions with a 12 h light/dark cycle, temperatures ranging from 22 to 24 °C, and humidity levels of 40% to 60% at the Central Animal Research Facility of Ras Al Khaimah Medical and Health Sciences University (RAKMHSU), UAE. They had constant access to tap water and were fed a standard rat pellet diet. After a week of acclimatization, the rats were randomly assigned to different experimental groups. Ethics approval for this study was obtained from the RAKMHSU Research and Ethics Committee (RAKMHSU-REC-114-2022/23-UG-M). The study underwent a comprehensive ethical review and adhered to all relevant guidelines for the humane and ethical treatment of animals in research. All procedures were carried out in strict accordance with the guidelines set by the appropriate regulatory authorities, with a primary focus on the principle of beneficence. In alignment with the 3Rs (Replacement, Reduction, and Refinement), we used small laboratory animals (Wistar rats) due to their significant genetic similarity to humans, employed a statistically recommended sample size of six per group, and applied methods designed to minimize pain, suffering, and distress, thereby enhancing the welfare of the animals involved.

### 2.3. Rationale for Dose Selection of Cisplatin, Silymarin and Dapagliflozin and Their Dissolution

The cisplatin dose was standardized and validated to induce hepatotoxicity in the rats [28]. We have earlier reported that 50 mg/kg of silymarin exerts good hepatoprotective effects [16]. The recommended daily dosage of dapagliflozin for its anti-diabetic effects in humans, as approved by the US FDA, is 10 mg. Using the body surface area ratio method outlined by Paget and Barnes, this human dosage was adjusted to an equivalent rat dosage of 0.9 mg/kg/day. Both the test drugs (silymarin and dapagliflozin) were dissolved separately in a 2% gum acacia solution and given orally.

### 2.4. Experimental Design

After recording their initial body weight, 30 adult female Wistar rats, aged 8–10 weeks, were randomly assigned to five different groups, with six rats in each group. The treatment protocol was administered daily for 45 days between 10 and 11 AM, outlined as follows:

Group I (Normal control): Normal saline (0.9% NaCl, 1 mL/kg; i.p. once every week) + 2% gum acacia (1 mL/kg/day; p.o) for 45 days

Group II (Negative control): Cisplatin-intoxicated hepatotoxic control rats (cisplatin 3 mg/kg; i.p. once every week) + 2% gum acacia (1 mL/kg/day; p.o) for 45 days

Group III (Positive control; Cisplatin + Silymarin): Cisplatin-intoxicated hepatotoxic rats (cisplatin 3 mg/kg; i.p. once every week) + Silymarin (50 mg/kg/day; p.o) for 45 days

Group IV (Test; Cisplatin + Dapagliflozin): Cisplatin-intoxicated hepatotoxic rats (cisplatin dose 3 mg/kg; i.p. once every week) + Dapagliflozin (0.9 mg/kg/day; p.o) for 45 days

Group V (Test; Cisplatin + Silymarin + Dapagliflozin): Cisplatin-intoxicated hepatotoxic rats (cisplatin 3 mg/kg; i.p. once every week) + Silymarin (50 mg/kg/day; p.o.) + Dapagliflozin (0.9 mg/kg/day; p.o) for 45 days

Body weight was recorded on a weekly basis throughout the experiment. On the 46th day, the rats were anesthetized with an intraperitoneal injection of ketamine (60 mg/kg) and xylazine (10 mg/kg). Blood glucose levels were measured by a glucometer using glucose oxidase–peroxide-reactive strips (Trister, Lewes, DE, USA) after obtaining fasting blood samples from the tail vein.

### 2.5. Collection of Blood and Serum Preparation

Using capillary tubes, blood was obtained from the retro-orbital plexus of veins in microcentrifuge tubes, following which the serum was separated from whole blood using a cooling centrifuge at 3000 rpm at 4 °C for 20 min.

### 2.6. Collection of the Liver and Its Gross Examination

Anesthetized animals were humanely euthanized using an overdose of anesthetics following blood collection. Subsequently, they were positioned supine on the operation table. A single incision was made using a surgical scalpel from the ventral thoracic to the abdominal wall to access the visceral cavities. Ascites and pleural effusion were then examined for their presence. The liver was carefully dissected from the surrounding abdominal muscles, fascia, visceral fats, and vasculature. A thorough gross morphological examination of the liver was conducted. Subsequently, the liver was rinsed with regular saline and placed on blotting paper to remove excess blood. Finally, it was preserved in 10% formalin for subsequent histopathological analysis.

### 2.7. Liver Function Test

The liver function test is a crucial aspect of medical diagnostics, providing valuable insights into the health and functionality of the liver. These tests play a pivotal role in detecting liver pathologies, monitoring ongoing conditions and assessing the therapeutic impact of various medications. Liver function test kits, designed for use with autoanalyzers, typically include reagents and calibrators necessary for measuring specific liver-related parameters—AST, ALT, TB, TP and albumin were assessed in the serum as per the standard protocols provided with the corresponding assay kits. Each of these parameters provides valuable information about different aspects of liver function. Each reagent is specific to the parameter being measured. The serum was mixed with the reagents provided in the liver function test kit. The prepared samples were loaded into the autoanalyzer, along with the necessary controls and calibrators. The autoanalyzer (Model-AviChem Mini; Manufacturer—Avicenna, Alliance Global, Dubai, UAE) performed the analysis by utilizing predefined protocols for each parameter. It measured the absorbance emitted by the reaction between the sample and the reagents. The autoanalyzer calculated the concentration of each parameter based on the obtained readings. The results were then generated and reported electronically.

### 2.8. Microscopic Evaluation of Liver Tissue for Qualitative Histopathology

Liver tissue samples were extracted from 1–2 rats per group and preserved in 10% formalin. A portion of each sample was then processed by cutting and dehydrating through a series of ethyl alcohol concentrations. The tissues were then cleared with xylene until transparent and embedded in molten paraffin wax. After 24 h, 6-micron-thick sections were cut using a microtome, mounted on albumenized glass slides, and labeled. These sections were de-waxed with xylene for 10 min, rehydrated through decreasing concentrations of ethyl alcohol (100%, 90%, 70%, 50%) for 2 min each, and finally in distilled water for 10 min. The sections were then stained with Harris hematoxylin for 5 min, rinsed in running tap water for 10 min, stained with 2% eosin for 2 min, and washed with 90% alcohol for 2 min, followed by 100% alcohol for another 2 min. They were cleared in 99.14% xylene, and 2–3 drops of DPX mountant were applied before placing coverslips to prevent drying. The slides were examined for morphological changes using a light microscope (Olympus BX53) at 400× magnification, and photomicrographs were taken for qualitative analysis.

### 2.9. Data Analysis

Data were analyzed using SPSS version 29, with normally distributed results expressed as mean ± standard deviation. A two-way analysis of variance (ANOVA) was performed, followed by a post hoc Tukey’s test for further comparison. Statistical significance was considered at a threshold of *p* < 0.05.

## 3. Results

### 3.1. Impact on Liver Function Test

**Figure 1:**
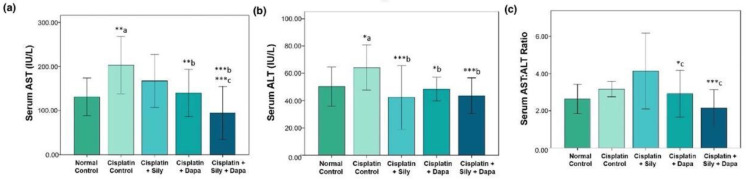
Effect on serum levels of-(**a**) AST, (**b**) ALT and (**c**) Ratio of AST and ALT; Sily—Silymarin, Dapa—Dapagliflozin; *** *p* < 0.001, ** *p* < 0.01, * *p* < 0.05; a—compared to normal healthy control group, b—compared to Cisplatin intoxicated hepatotoxic control group, c—compared to silymarin treated hepatotoxic group.

**Figure 2:**
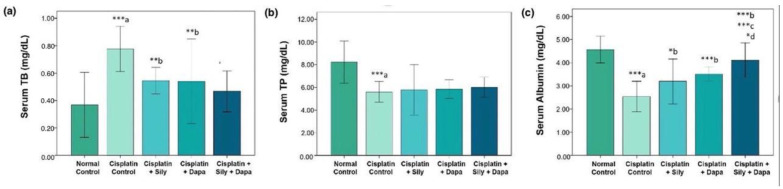
Effect on serum levels of-(**a**) Total bilirubin, (**b**) Total protein and (**c**) Albumin; Sily—Silymarin, Dapa—Dapagliflozin; *** *p* < 0.001, ** *p* < 0.01, * *p* < 0.05; a—compared to normal healthy control group, b—compared to Cisplatin intoxicated hepatotoxic control group, c—compared to silymarin treated hepatotoxic group, d—compared to dapagliflozin treated hepatotoxic group.

The liver function tests indicated a significant increase (*p* < 0.001) in serum levels of AST (*p* = 0.002), ALT (*p* = 0.041), and TB (*p* < 0.001) in the hepatotoxic control group compared to the normal control. Additionally, there was a substantial decrease in total protein (TP) (*p* < 0.001) and albumin (*p* < 0.001) in the hepatotoxic control group. Conversely, treatment with dapagliflozin in hepatotoxic rats resulted in a significant reduction in ALT (*p* = 0.017), AST (*p* = 0.006), and TB (*p* = 0.004), along with a marked increase in albumin (*p* < 0.001) compared to the cisplatin-intoxicated hepatotoxic control group. Silymarin also showed a significant reduction in ALT (*p* < 0.001) and TB (*p* = 0.005), and a notable increase in albumin (*p* = 0.023) relative to the cisplatin-intoxicated hepatotoxic control. Interestingly, the combination of dapagliflozin and silymarin led to a significant decrease (*p* < 0.001) in the serum levels of ALT, AST, and TB, and a significant increase in albumin levels (*p* < 0.001) compared to the cisplatin-intoxicated hepatotoxic control group. Moreover, the group treated with both silymarin and dapagliflozin demonstrated a significant reduction in AST (*p* < 0.001) and a notable increase in albumin (*p* < 0.001) compared to the group treated with silymarin alone. Additionally, we observed a significant elevation (*p* = 0.042) in albumin levels for hepatotoxic rats treated with a combination of silymarin and dapagliflozin compared to rats treated with dapagliflozin alone (Figure 1 and Figure 2).

### 3.2. Effect on Body Weight and Fasting Blood Glucose Levels

**Figure 3:**
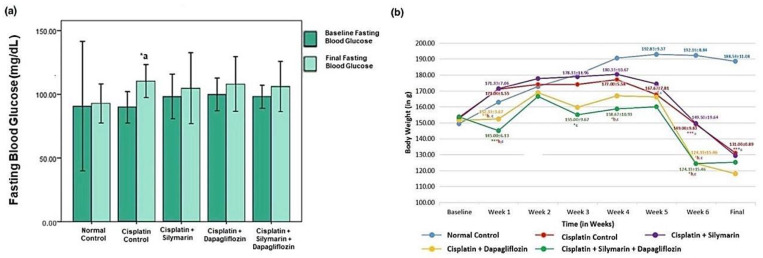
Effect on-(**a**) Fasting Blood Glucose and (**b**) Body Weight; *** *p* < 0.001, ** *p* < 0.01, * *p* < 0.05; a—compared to normal control, b—compared to Cisplatin intoxicated hepatotoxic control, c—compared to silymarin treated hepatotoxic group. n = 6/group. All values on the *y*-axis depict the mean. Error bars +/− 2SD.

At baseline, there was no significant reduction in the body weight of the experimental rats. However, a notable reduction (*p* < 0.05) in body weight was detected in the hepatotoxic rats that were administered dapagliflozin or a combination of dapagliflozin and silymarin compared to normal healthy control rats during the entire treatment duration (Figure 3a). Starting from week 5 onwards, there was a notable decrease (*p* < 0.001) in the weight of the rats intoxicated with cisplatin in comparison to the normal healthy control. However, we did not observe any significant changes in the body weight of the rats treated with dapagliflozin alone and in combination with silymarin compared to the cisplatin-intoxicated hepatotoxic control rats from week 5 onwards (Figure 3a).

A notable reduction (*p* < 0.05) in fasting blood glucose was observed in the cisplatin-intoxicated hepatotoxic control (110.33 ± 6.47) when compared to the normal healthy control (92.83 ± 7.62). No significant variation (*p* > 0.05) was found in the fasting blood glucose levels among the other experimental groups during screening and at the experiment’s conclusion (Figure 3b).

### 3.3. Effect on Mortality 

**Figure 4:**
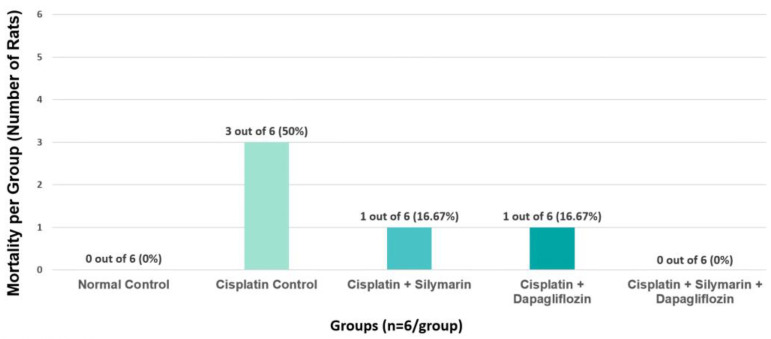
Effect of on Group-Specific Mortality.

In the cisplatin-intoxicated hepatotoxic control group, the mortality rate was observed to be 50%. In contrast, the mortality rate was 16.67% in both the silymarin-only and dapagliflozin-only treatment groups. Notably, there were no instances of mortality in the normal healthy control rats and the groups receiving combination treatment with dapagliflozin and silymarin (Figure 4). Due to the significant mortality in Group II (Negative control) mainly due to cisplatin treatment, we requested and received approval from our Institutional Animal Ethics Committee to use additional animals from the Central Animal Research Facility, allowing us to continue the experiments with an adequate sample size.

### 3.4. Impact on Gross Morphological Examination of the Liver and Hepatocellular Architecture

During gross morphological examination, the liver of the normal healthy control group was observed to be dark reddish-brown with a smooth and soft texture. Conversely, the livers of the cisplatin-induced hepatotoxic control rats showed a somewhat pale coloration. The gross morphological changes in the cisplatin-induced hepatotoxic control rats were not as pronounced as those typically seen in models of ethanol, paracetamol, rifampicin, or carbon tetrachloride-induced hepatotoxicity.

**Figure 5:**
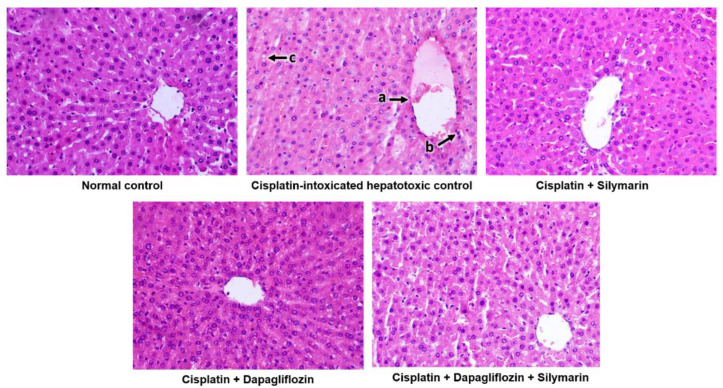
Qualitative histopathological examination of liver; Representative images of liver tissues stained with H&E seen at 400× magnification under the light microscope; a—Mildly dilated and congested central vein, b—Mononuclear cell infiltration around the central vein, c—Focal hepatocyte cytoplasmic vacuolation.

The normal control group demonstrated the typical architecture of hepatic cells, with hepatocytes organized in concentric hepatic cords surrounding the central vein. In contrast, the cisplatin-intoxicated control group exhibited mildly dilated and congested central vein, mononuclear cell infiltration around the central vein, and focal hepatocyte cytoplasmic vacuolation. Meanwhile, the silymarin-treated hepatotoxic rats displayed only mildly dilated central vein. Surprisingly, hepatotoxic rats treated with dapagliflozin alone and its combination with silymarin exhibited a normal architecture of hepatic cells similar to that of the normal control (Figure 5).

## 4. Discussion

The present study highlights the potential of dapagliflozin as a hepatoprotective agent, either alone or in combination with silymarin. Our study utilized a comprehensive approach, a multifaceted approach to demonstrate the hepatoprotective benefits of dapagliflozin in countering cisplatin-induced liver damage. Results of this study not only enhance our comprehension of the diverse impacts of dapagliflozin but also present promising avenues for therapeutic intervention to mitigate the adverse effects associated with cisplatin-based chemotherapy, thereby enhancing the safety and efficacy of cancer treatment. The research findings provide an in-depth understanding of the liver-protective effects of dapagliflozin and silymarin in relation to cisplatin-induced liver damage. These effects are evidenced by notable changes in the liver function and the distinct histological modifications. Clinical use of cisplatin has been linked to elevated serum levels of AST, ALT, ALP, and LDH, suggesting a potential risk of liver injury associated with cisplatin use [29].

One of the studies reported that cells treated with cisplatin and overexpressing CYP2E1 exhibited a notable rise in oxidative stress in comparison to cells lacking this enzyme [11]. The introduction of oxygen radicals into the original drug could lead to the formation of a highly toxic reactive molecule. One of the studies indicated that cisplatin produced not only ROS but also heme-derived ROS mediated by the action of CYP450 [9]. Consequently, the heightened formation of hydrogen peroxide, superoxide radicals, and hydroxyl radicals were responsible for tissue damage, apoptosis, liver failure, and acute renal failure [30].

Cisplatin accumulation in liver cells results in hepatotoxicity, primarily driven by heightened oxidative stress and inflammation [31]. Additionally, cisplatin impacts the tumor-suppressor protein p53 by producing ROS, leading to apoptosis [32]. Cisplatin treatment is associated with adverse effects such as a loss of liver histoarchitecture, positive caspase-3 reactions, a decline in GSH, and an elevation in MDA levels [33]. Since oxidative stress is pivotal in hepatotoxicity induction, the utilization of antioxidants can significantly mitigate cisplatin-induced toxicity [23].

The liver function test results provide compelling evidence of the hepatoprotective effects of dapagliflozin and silymarin in rats with cisplatin-induced hepatotoxicity. Cisplatin exposure significantly elevated serum levels of ALT, AST, and TB, which is indicative of liver damage, while concurrently decreasing TP and albumin levels. However, treatment with dapagliflozin led to a substantial reduction in ALT, AST, and TB, along with a significant elevation in albumin levels, compared to the hepatotoxic control group. Similar hepatoprotective effects were observed with silymarin, consistent with the previous studies demonstrating its efficacy in mitigating liver damage [16,34]. The combined therapy of silymarin and dapagliflozin resulted in a more pronounced decrease in ALT, AST, and TB, and a more substantial rise in albumin levels in comparison to either of them. This suggests a potential synergism between dapagliflozin and silymarin in ameliorating cisplatin-induced hepatotoxicity, warranting further investigation into the underlying mechanisms. Comparing dapagliflozin with silymarin, both compounds demonstrated significant hepatoprotective effects, but dapagliflozin appeared to be more effective in reducing AST levels. This finding is noteworthy as AST is a crucial enzyme reflecting mitochondrial damage, and the reduction in its levels by dapagliflozin may indicate a specific protective mechanism against mitochondrial injury [35]. Additionally, the combination displayed a further reduction in AST levels compared to silymarin alone, implying a potential synergistic effect. The significant increase in albumin levels in the combination group, compared to rats treated with dapagliflozin alone, suggests an enhancement of the liver synthetic function with the addition of silymarin. This emphasizes the importance of exploring combination therapies for hepatoprotection. SGLT-2 inhibitors have been reported to exhibit the capacity to stimulate lipolysis, ketone synthesis, mitochondrial formation and autophagy. Concurrently, they mitigate lipid synthesis, oxidative stress, endoplasmic reticulum stress, the renin–angiotensin–aldosterone system, apoptosis, and fibrosis [36]. A clinical trial consisting of 156 patients demonstrated that the use of SGLT-2 inhibitors not only enhanced blood glucose control in individuals with NAFLD and T2DM but also significantly reduced visceral fat area and body weight [37]. Over a 24-week period of treatment with SGLT-2 inhibitor, a reduction in the liver hardness and fibrosis stage markers were noted [38]. Reports indicate that SGLT-2 inhibitors notably boost autophagy in liver macrophages through the AMPK/mTOR signaling axis, alleviating liver damage in T2DM mice with NAFLD [39]. Increasing clinical evidence now supports the effective role of SGLT-2 inhibitors in liver protection for individuals with T2DM.

Interestingly, despite dapagliflozin being an antidiabetic agent, no prominent changes were noted in fasting blood glucose levels among the experimental groups. This result suggests that the hepatoprotective effects of dapagliflozin observed in this study may be independent of its glucose-lowering effects. The reduction in body weight observed in dapagliflozin-treated hepatotoxic rats may be attributed to factors other than glucose regulation, such as changes in fluid balance or metabolic alterations [40].

Hepatotoxicity has been observed in patients receiving low doses of cisplatin due to its accumulation in the liver. This includes the effacement of hepatic cords, focal inflammatory pathologies, and necrosis, as indicated by studies [14]. Notable histological findings in cisplatin-induced liver toxicity include sinusoidal dilatations, hepatocellular vacuolation, and cytoplasmic changes around the central vein [41].

Microscopic histopathological analysis of the liver tissue corroborated the liver-protective effects of dapagliflozin and silymarin. Rats treated with dapagliflozin, silymarin, or their combination exhibited liver structures that closely resembled those of the normal control group. These observations are consistent with liver function test results, underscoring the potential therapeutic advantages of dapagliflozin and silymarin treatments.

Dapagliflozin treatment has been shown to improve steatohepatitis in diabetic mice undergoing experimental steatohepatitis due to the inhibition of oxidative stress and decreased activity of the NLRP3 inflammasome. The amelioration of cisplatin-induced decline in liver function and normalization of hepatocellular architecture may be attributed to the inhibition of oxidative stress and reduced activity of the NLRP3 inflammasome by dapagliflozin alone and in combination with silymarin [42].

Dapagliflozin, an SGLT2 inhibitor, is known for its antidiabetic effects. The absence of significant differences in fasting blood glucose levels among groups suggests that dapagliflozin’s hepatoprotective effects are not mediated by glycemic control in this model. Notably, a decrease in body weight was observed in both the dapagliflozin-treated and combination-treated groups, prompting further investigation into potential metabolic effects. Several clinical studies have reported that dapagliflozin monotherapy among type 2 diabetic patients decreases the reabsorption of filtered glucose and enhances urinary glucose excretion. Consequently, this results in the loss of calories through glucose excretion in urine and initiates a negative energy balance, ultimately causing weight reduction over time [43,44]. One study indicated that dapagliflozin might also decrease fat mass, thereby contributing to weight loss. Although the precise mechanism remains incompletely understood, it likely involves alterations in energy metabolism and hormonal regulation [45].

In the present study, the mortality of 50% in the cisplatin-intoxicated hepatotoxic control group underscores the severity of cisplatin toxicity and its potentially lethal consequences. This finding is consistent with the evident toxic effects of cisplatin, which can lead to organ failure and ultimately death in experimental animals. However, the administration of silymarin alone and dapagliflozin alone resulted in a notable reduction in mortality to 16.67% in both treatment groups. This indicates the potential therapeutic benefits of silymarin, a flavonoid with known antioxidant and hepatoprotective properties, as well as dapagliflozin, a sodium–glucose cotransporter 2 (SGLT2) inhibitor primarily used in the management of type 2 diabetes, in mitigating the toxic effects of cisplatin. The most striking observation is the complete absence of mortality in the groups receiving combination treatment with dapagliflozin and silymarin. This finding suggests a potential therapeutic benefit with the use of these two agents in conferring protection against cisplatin-induced toxicity. Further investigation into the underlying mechanisms responsible for this therapeutic effect could provide valuable insights into novel therapeutic strategies for mitigating cisplatin-induced mortality.

In the study by Hassan et al., the authors investigated the acute hepatotoxicity caused by a single dose of cisplatin at 2 mg/kg in rabbits [46]. They further demonstrated that L-Carnitine, known for its antioxidant properties, effectively mitigated this acute hepatotoxicity, as indicated by a reduction in hepatic injury biomarkers. In contrast, our study focused on chronic hepatotoxicity induced by cisplatin through a more clinically relevant dosing regimen. We administered cisplatin at a repetitive dose of 3 mg/kg/week over six weeks, resulting in a treatment duration of 45 days. This dosing pattern mimicked the cyclic administration of chemotherapy in clinical settings and allowed the liver to have intermittent “chemo-free” periods, giving it time to recover from the toxic effects of cisplatin. Consequently, the hepatotoxic effects observed in our study were less severe than those reported by Hassan et al., likely due to the liver’s inherent regenerative capacity when given sufficient recovery time [46]. Regarding the hepatoprotective effects, our study found that dapagliflozin, much like L-Carnitine in the Hassan et al. study, significantly ameliorated hepatic injury biomarkers [46]. Dapagliflozin’s efficacy in reducing cisplatin-induced hepatotoxicity can be attributed to its ability to modulate oxidative stress and inflammation, similar to the mechanism proposed for L-Carnitine. However, dapagliflozin may offer certain advantages in specific patient populations. For instance, dapagliflozin is an established treatment in patients with type 2 diabetes mellitus (T2DM) and has been shown to have cardioprotective and renoprotective effects. In T2DM patients undergoing cisplatin-based chemotherapy, dapagliflozin could provide an additional chemoprotective benefit, particularly in mitigating cisplatin-induced hepatotoxicity. This dual benefit makes dapagliflozin a potentially more suitable option for this patient group compared to L-Carnitine, which is primarily prescribed for its antioxidant properties and is widely used in chronic kidney disease patients. Moreover, based on our findings and the literature, we hypothesize that a combination therapy of L-Carnitine and dapagliflozin could be a gold-standard approach in patients undergoing cisplatin-based chemotherapy. This combination could potentially offer synergistic protection against cisplatin-induced hepatotoxicity. However, further studies are warranted to explore the safety, efficacy, and potential drug interactions of this combination therapy. Although both L-Carnitine and dapagliflozin have demonstrated significant potential in mitigating cisplatin-induced hepatotoxicity, dapagliflozin may have added benefits in patients with T2DM undergoing chemotherapy.

Our study has several notable strengths, including a comprehensive approach that integrates biochemical and histopathological analyses to thoroughly assess liver function. We investigated the possibility of repurposing the well-known anti-diabetic drug dapagliflozin, administered at a dose of 0.9 mg/kg/day, which corresponds to the standard human dosage of 10 mg/day while maintaining safety considerations. However, the study has some limitations including factors like a limited sample size and the employment of an animal model for cisplatin-induced hepatotoxicity that does not include cancer. This may affect the applicability of the results to human physiology and pathophysiology, limiting their generalizability to clinical settings. Despite these limitations, our research provides valuable insights into the hepatoprotective effects of dapagliflozin alone and in combination with silymarin to combat cisplatin-induced liver toxicity. Overcoming these limitations would improve the strength and relevance of our findings.

## 5. Conclusions

Our study revealed the notable liver-protective effects of dapagliflozin and silymarin in a cisplatin-induced hepatotoxicity model in Wistar rats. The combined therapy exhibited beneficial effects across diverse parameters, almost exceeding the effects of dapagliflozin and silymarin when used separately. The promising results of our study indicate that the co-administration of dapagliflozin (SGLT2i) and silymarin presents a potentially effective treatment strategy for mitigating cisplatin-induced hepatotoxicity. The comprehensive impact observed, including improvements in liver function and hepatocellular morphology, underscores the potential of this combination as a holistic intervention targeting various aspects of hepatic injury. The investigation into the underlying molecular mechanisms and the long-term safety and efficacy of dapagliflozin in hepatotoxicity models remains a crucial area for future research, paving the way for potential clinical translation. Overall, this research significantly contributes to the growing body of knowledge on hepatoprotective agents and their potential application in mitigating chemotherapy-induced liver damage. This study opens avenues for exploring novel therapeutic strategies in the management of drug-induced liver injury, with potential implications for clinical applications. Exploring the potential for translating dapagliflozin and silymarin in managing cisplatin-induced hepatotoxicity holds promise for enhancing clinical outcomes in individuals undergoing cisplatin-based chemotherapy.

## Data Availability

All data arising from this study are included within the article.

## Abbreviations

Dapa—Dapagliflozin; Sily—Silymarin; ALT—alanine transaminase; AST—aspartate transaminase; TB—Total bilirubin; TP—Total protein; ROS—Reactive oxygen species; NLRP3—nucleotide-binding domain, leucine-rich–containing family, pyrin domain–containing-3; MDA—Malondialdehyde; SGLT2—Sodium-glucose co-transporter-2; p.o—Per oral; i.p—Intraperitoneal; H&E—Hematoxylin and Eosin; DPX—Dibutylphthalate polystyrene xylene; NAFLD—Non-alcoholic fatty liver disease; T2DM—Type 2 diabetes mellitus; SPSS—Statistical package for social sciences; RAKMHSU—RAK Medical and Health Sciences University; REC—Research and Ethics Committee; US—United States; FDA—Food and Drug Administration.
